# Intestinal protozoa in hospitalized under-five children with diarrhoea in Nampula – a cross-sectional analysis in a low-income setting in northern Mozambique

**DOI:** 10.1186/s12879-021-05881-7

**Published:** 2021-02-23

**Authors:** Adilson Fernando Loforte Bauhofer, Idalécia Laurinda Carlos Cossa-Moiane, Selma Domingos Amadeu Marques, Esperança Lourenço Alberto Mabandane Guimarães, Benilde António Munlela, Elda Muianga Anapakala, Jorfélia José Chiláule, Marta Cassocera, Jerónimo Souzinho Langa, Assucênio Chissaque, Júlia Assiat Monteiro Sambo, Lena Vânia Manhique-Coutinho, Diocreciano Matias Bero, Timothy Allen Kellogg, Luzia Augusta Pires Gonçalves, Nilsa de Deus

**Affiliations:** 1 Instituto Nacional de Saúde (INS), Maputo, Mozambique; 2 Instituto de Higiene e Medicina Tropical, Universidade Nova de Lisboa, Lisboa, Portugal; 3 Institute of Tropical Medicine, Antwerp, Belgium; 4 Centro de Biotecnologia, Universidade Eduardo Mondlane, Maputo, Mozambique; 5 Institute for Global Health Sciences, University of California San Francisco, California, USA; 6 Global Health and Tropical Medicine, Instituto de Higiene e Medicina Tropical, Universidade Nova de Lisboa, Portugal and Centro de Estatística e Aplicações da Universidade de Lisboa, Lisboa, Portugal; 7 Departamento de Ciências Biológicas, Universidade Eduardo Mondlane, Maputo, Mozambique

**Keywords:** Diarrhoea, Children, Intestinal Protozoa, Related factors, Low-income setting, Nampula province, Mozambique

## Abstract

**Background:**

In Mozambique, infection by intestinal parasites is reported all over the country. However, infection in children with diarrhoea is mostly focused in the southern region of Mozambique. This work aims to determine the frequency and potential risk factors for infection by *Cryptosporidium* spp., *Giardia lamblia,* and *Entamoeba histolytica* in children under-five years hospitalized with diarrhoea in *Hospital Central de Nampula*, northern Mozambique.

**Methods:**

A cross-sectional hospital-based surveillance was conducted between March 2015 and January 2018 in children admitted with diarrhoea in *Hospital Central de Nampula*. Sociodemographic information was obtained through semi-structured interviews applied to the children’s caregivers. A single stool sample was collected from each child to detect antigens from *Cryptosporidium* spp., *G. lamblia,* and *E. histolytica* using an immune-enzymatic technique. Crude and adjusted odds ratios (with 95% Confidence Intervals) were obtained by logistic regression models to identify factors associated with infection by *Cryptosporidium* spp. and *G. lamblia*.

**Results:**

The median age and interquartile intervals of our sample population was 12 months (8–20). Intestinal protozoa were detected in 21.4% (59/276). *Cryptosporidium* spp. was the most common protozoa (13.9% - 38/274), followed by *G. lamblia* (9.1% - 25/274) and *E. histolytica* (0.4% - 1/275). Children with illiterate caregiver’s (*p*-value = 0.042) and undernourished (*p*-value = 0.011) were more likely to be infected by *Cryptosporidium* spp. *G. lamblia* was more common in children living in households with more than four members (p-value = 0.039). *E. histolytica* was detected in an eleven month’s child, co-infected with *Cryptosporidium* spp. and undernourished.

**Conclusion:**

*Cryptosporidium* spp. and *Giardia lamblia* were the most common pathogenic intestinal protozoa detected in children with diarrhoea hospitalized in the *Hospital Central de Nampula*. Our findings obtained highlight the importance of exploring the caregiver’s education level, children’s nutritional status for infections with *Cryptosporidium* spp., and living conditions, namely crowded households for infections with *G. lamblia* in children younger than five years.

## Background

Diarrhoea is the second leading cause of death in children under five years [1]. Diarrhoea can be caused by bacteria, viruses, and parasites [2–4]. Among parasites, the intestinal protozoa *Cryptosporidium* spp. is the most attributable to moderate-to-severe diarrhoea in children younger than five in sub-Saharan Africa and Asia [3]. The second and third most common intestinal protozoa identified in children with diarrhoea are *Giardia lamblia* and *Entamoeba histolytica*, respectively [5–7]*.*

Transmission of intestinal protozoa occurs by the ingestion of contaminated food and water or by direct contact with infected individuals or animals [8, 9]. Host and environmental characteristics such as age, nutritional status, access to treated water, animal contact, and, population density are important factors in the occurrence of parasites infections [10–14].

Mozambique is a low-income country, with reports of intestinal parasites infections in children with diarrhoea, mostly in the southern region, having identified a frequency of 16.1% in Maputo city and 14.4% in Manhiça district, using microscopic diagnostic techniques [3, 7, 15, 16]. Using a more sensitive technique, the Enzyme-Linked Immunosorbent Assay (ELISA), a second study also in the Manhiça district reported the frequency of *Cryptosporidium* spp.*, G. lamblia*, and *E. histolytica/dispar* in children with diarrhoea by 18.7, 17.2, and 10.2%, respectively [7].

A national survey in school-age children identified Nampula - a northern province of Mozambique - as one of the most affected by parasites infections [17]. A second study conducted in *Hospital Central de Nampula* (HCN) observed *G. lamblia* as the most common parasite, followed by *Cryptosporidium* spp. in a pooled estimation in children younger than five years with either diarrhoea, malnutrition, or Human Immunodeficiency Virus (HIV) [18]. However, parasites infections in children with only diarrhoea are not reported, neither risk factors for this group [18].

A pooled analysis of a surveillance system in four provinces of Mozambique indicated Nampula province as the one with the highest risk of infection for *Cryptosporidium* spp. in children younger than 15 years [5]. Nampula province has poor health indicators, with recurrent reports of cholera outbreaks [19]. The previous results from the surveillance system (*Vigilância Nacional de Diarreias* – ViNaDia or National Surveillance of Diarrhoea) failed to indicate risk factors for protozoans in each province [5]. Therefore, this analysis aims to determine the frequency of intestinal protozoa infections and associated risk factors in children younger than five years admitted with diarrhoea in *Hospital Central de Nampula*, in the Northern region of Mozambique.

## Methods

### Study design, site, population

Hospital-based, cross-sectional surveillance (ViNaDia) was conducted from March 2015 to January 2018 in *Hospital Central de Nampula* (HCN), Nampula province, located in a low-income area of the North region of Mozambique. This hospital was selected for the following reasons: HCN is the provincial and regional referrall hospital, has an inpatient paediatric service, and a reliable system for collection, storage, and transportation of stool samples to *Instituto Nacional de Saúde* (INS) – Mozambique.

Children aged 0–59 months hospitalized in the HCN paediatric services with diarrhoea, defined as the passage of three or more loose or liquid stools in 24 h were included [20], children who could provide a stool sample and those whose caregivers consented their participation in the ViNaDia were eligible to participate. Children with nosocomial diarrhoea were ineligible to participate in the ViNaDia.

Children overweight (+ 2 < z ≤ + 5) or with outlier Z-scores values were excluded from the nutritional status analysis [21].

### Sample size calculation

OpenEpi [22] was used to calculate the minimum sample size necessary to estimate the prevalence of parasites with 95% confidence interval (CI), design effect 1, desired precision of 5.0%, and an estimated frequency of 18.7% for *Cryptosporidium* spp.*,* 17.2% for *Giardia lamblia*, and 10.2% for *Entamoeba histolytica* from a previous study conducted in Mozambique in children aged 0–59 months with diarrhoea [7]. Based on the first estimate (18.7%) that provides the higher value of the sample size and a 10% non-response expected rate, the sample size was 258 children.

### Variables of analysis and anthropometric measurements

Sociodemographic and epidemiological data were previously obtained at the health facility using semi-structured interviews with the children’s caregivers. Some of the variables included information such as sex, age, history of animal contact defined as having close contact with an animal or their excrements in areas where the child circulated. Children’s weight was obtained in the clinical records or by scaling. For children under two years, weight was measured while the child was lying down, while the weight for children aged two years or older were measured standing. The anthropometric indicator, weight-for-age Z-score (WAZ) was calculated to identify underweight status using WHO software Anthro version 3.2.2 for children younger than five years. Nutritional status was classified as well-nourished (− 2 ≤ z ≤ + 2) and undernourished (− 6 ≤ z < − 2) [21].

### Sample collection and handling

A single stool sample from each child was collected in a polyester flask and stored in − 20 °C until shipment to the Laboratory of Parasitology (national reference laboratory) of INS in Maputo (capital of Mozambique) for laboratory diagnosis. Sample shipment to Maputo was made once a week during the recruitment period.

### Laboratory diagnosis

Intestinal protozoa diagnosis was performed using an individual Enzyme-Linked Immunosorbent Assay (ELISA) (TechLab, Inc., Blacksburg, VA, USA) according to manufacturer recommendations to detect antigens of *Cryptosporidium* spp. oocysts, *G. lamblia* cysts, and *E. histolytica* cysts from stool samples. The sensitivity of the kits range from 97 to 100%, and the specificity range from 95 to 100% [23–25].

### Data management and statistical analysis

To minimize entry errors, survey data were entered twice into a database using Epi Info™ 3.5.1 (Centers for Disease Control and Prevention, Atlanta, 2008) followed by data comparison and inconsistencies resolution if necessary.

IBM SPSS software (International Business Machines Corporation Statistical Package for the Social Science, Armok, NY: IBM Corp, 2011, version 26.0) was used to analyse data. Descriptive statistics were used to describe sample characteristics. Proportions and corresponding Wilson 95% CIs were estimated for each infection (by *Cryptosporidium* spp., *G. lamblia,* and *E. histolytica*) [26]. Cross tabulations were constructed between dependent (the three mentioned infections), and independent variables (e.g., sex, caregiver’s education level, household members with a cut-off of five members). Crude and adjusted odds ratios were estimated through simple and multiple logistic regression models using as dependent variables infection by *Cryptosporidium* spp. and *G. lamblia*. Independent variables with *p*-values ≤0.2 in the simple logistic regression and potential confounders were included in the multiple logistic regression models to obtain adjusted odds ratio. Hosmer and Lemeshow test was used to assess the fit of the multiple logistic regression models. Pairwise deletion procedure was used to handle missing data in the inferential analysis. *P*-value of less than 0.05 was considered statistical significant.

## Results

During the analysis period, 276 children admitted at HCN were included. However, 99.3% (274/276) of the children provided sufficient stool amounts to obtain results for *Cryptosporidium* spp. and *G. lamblia* and, 99.6% (275/276) for *E. histolytica*. Overall, 98.9% (273/276) of the children had enough stool amounts to obtain results for the three protozoans.

### Sample characteristics

Among the included children, 55.8% (154/276) were male; 48.6% (134/276) were less than 12 months of age; 46.0% (127/276) had caregivers with a secondary or greater educational level. Also, 64.5% (178/276) were living in a household with more than four members (Table 1). Animal contact was reported in 46% (127/276); 51.1% (141/276) drank water from the public tap, and 63.6% (171/276) were well-nourished for WAZ (Table 1).

**Table 1:** Characteristics of children hospitalized with diarrhoea in HCN, March 2015 to January 2018, Nampula, Mozambique

Characteristics	*N* = 276	%
*Sex*		
Male	154	55.8
Female	122	44.2
*Age categorized (in months)*		
0–11	134	48.6
12–23	93	33.7
24–59	49	17.8
*Caregiver Education Level*		
Illiterate	42	15.2
Primary	104	37.7
Secondary/Above	127	46.0
Unknown/Missing	3	1.1
*Number of Members in the Child Household*		
< 5	76	27.5
≥ 5	178	64.5
Unknown/Missing	22	8.0
*Animal contact*		
No	148	53.6
Yes	127	46.0
Unknown/Missing	1	0.4
*Drinking-Water Source*		
Well	58	21.0
Piped water	74	26.8
Public tap	141	51.1
Others	2	0.7
Unknown/Missing	1	0.4
*Underweight (Weight-for-age Z-Score)*		
No	171	63.6
Yes	79	29.4
Unknown/Missing	19	7.1

### The overall frequency of intestinal protozoa

Infection by any enteric protozoa was seen in 21.4% (59/276; 95% CI: 17.0–26.6) of the children. *Cryptosporidium* spp. was the most common protozoa at 13.9% (38/274; 95% CI: 10.3–18.5), followed by *G. lamblia* at 9.1% (25/274; 95% CI: 6.3–13.1) and *E. histolytica* at 0.4% (1/275; 95% CI: 0.1–2.0).

Positive children for *Cryptosporidium* spp. and *G. lamblia* had a median age (in months) and interquartile interval of 15 (10–24) and 11 (8–20), respectively.

Co-infection between two protozoa was observed in 1.8% (5/273) of children. The most and least common co-infections were *Cryptosporidium* spp./*G. lamblia* 1.4% (4/273) and, *Cryptosporidium* spp./*E. histolytica* 0.4% (1/273), respectively.

### Factors related to infection by *Cryptosporidium* spp., *Giardia lamblia*, and *Entamoeba histolytica*

Children whose caregivers had primary education level were less likely to be infected by *Cryptosporidium* spp. (Adjusted Odds Ratio: 0.290, 95% CI: 0.091–0.916) compared with the illiterates. Underweighted children were 2.7 times more likely of being infected by *Cryptosporidium* spp. compared to well-nourished children (p-value = 0.011; 95% CI: 1.261–5.798) (Table 2).

**Table 2:** Characteristics, frequencies, crude and adjusted odds ratio for infection by *Cryptosporidium spp. and Giardia lamblia*, in children hospitalized with diarrhoea in HCN, March 2015 to January 2018, Nampula, Mozambique

Characteristics		***Cryptosporidium spp.***					***Giardia lamblia***				
	***N*** = 274	n (%)	*P*-value	Crude OR (95% CI)	*P*-value	Adjusted OR (95% CI)	n (%)	*P*-value	Crude OR (95% CI)	*P*-value	Adjusted OR (95% CI)
*Sex*											
**Male**	153	16 (10.5)		1		1	14 (9.2)	0.986	1.007 (0.440–2.306)		
**Female**	121	22 (18.2)	0.069	1.903 (0.951–3.809)	0.045	2.157 (1.017–4.572)	11 (9.1)		1		
*Age categorized (in months)*			0.642					0.173		0.332	
**0–11**	134	21 (15.7)	0.375	1.598 (0.567–4.506)			8 (6.0)		1		1
**12–23**	92	12 (13.0)	0.652	1.290 (0.426–3.903)			10 (10.9)	0.187	1.921 (0.728–5.069)	0.227	1.834 (0.686–4.904)
**24–59**	48	5 (10.4)		1			7 (14.6)	0.071	2.689 (0.919–7.870)	0.191	2.224 (0.671–7.367)
*Caregiver Education Level*			0.075		**0.042**			0.774			
**Illiterate**	42	8 (19.0)		1		1	4 (9.5)		1		
**Primary**	103	8 (7.8)	0.056	0.358 (0.15–1.028)	**0.035**	**0.290 (0.091–0.916)**	11 (10.7)	0.834	1.136 (0.340–3.791)		
**Secondary/Above**	126	22 (17.5)	0.816	0.899 (0.367–2.205)	0.803	0.883 (0.331–2.351)	10 (7.9)	0.748	0.819 (0.243–2.763)		
**Unknown/Missing**	3	–					–				
*Number of household member*											
**< 5**	76	12 (15.8)		1			2 (2.6)		1		1
**≥ 5**	176	21 (11.9)	0.406	0.723 (0.336–1.555)			21 (11.9)	**0.032**	**5.013 (1.145–21.947)**	**0.039**	**4.762 (1.083–20.935)**
**Unknown/Missing**	22	–					–				
*Animal contact*											
**No**	146	24 (16.4)	0.200	1.588 (0.783–3.220)	0.091	1.960 (0.899–4.273)	12 (8.2)		1		
**Yes**	127	14 (11.0)		1		1	13 (10.2)	0.565	1.273 (0.559–2.901)		
**Unknown/Missing**	1	–					–				
*Drinking-Water Source*			0.373					0.855			
**Well**	56	5 (8.9)		1			5 (8.9)	0.925	1.054 (0.353–3.142)		
**Piped water**	74	10 (13.5)	0.421	1.594 (0.512–4.957)			8 (10.8)	0.582	1.303 (0.508–3.344)		
**Public tap**	141	22 (15.6)	0.225	1.886 (0.677–5.255)			12 (8.5)		1		
**Others**	2	1 (50.0)	0.119	10.200 (0.550–189.125)			0 (0.0)		–		
**Unknown/Missing**	1	–					–				
*Underweight (Weight-for-Age Z-Score)*											
**No**	170	19 (11.2)		1		1	16 (9.4)		1		
**Yes**	78	17 (21.8)	**0.030**	**2.215 (1.079–4.544)**	**0.011**	**2.704 (1.261–5.798)**	9 (11.5)	0.606	1.255 (0.529–2.980)		
**Unknown/Missing**	19	–					–				

Children living in a household with more than four members were 4.7 times more likely to be infected by *G. lamblia* compared with children from households with fewer members (*p*-value = 0.039; 95% CI: 1.083–20.935) (Table 2).

*E. histolytica* was detected in a single male child, 11 months old, co-infected by *Cryptosporidium* spp*.* and undernourished. Due to the low positivity, logistic regression models were not built for infection by *E. histolytica*.

## Discussion

Findings from this analysis suggest that pathogenic intestinal protozoa infection is a public health problem in this setting, with one in five children with diarrhoea infected with at least one intestinal protozoa. The overall frequency of parasitic infections (21.4%) is higher than reported in the southern region of Mozambique (14.4 and 16.1%) [15, 16]. Differences observed may be due to the fact we used a more sensitive technique compared to microscopy used in the previous studies [27, 28].

*Cryptosporidium* spp. was the most common parasite (13.9%), followed by *G. lamblia* (9.7%) and *E. histolytica* (0.4%). Using the same diagnostic technique, a rural setting in the Manhiça district showed higher frequencies for *Cryptosporidium* spp. (18.7%) and *G. lamblia* (17.2%) among children with diarrhoea [7]. *E. histolytica* was observed in less than 1 % of the overall samples, similar to the reported in Tanzania, a country near Nampula province [6]. However, in the Manhiça district, the frequency of *E. histolytica/dispar* was much higher, 10.2%, suggesting different geographic distribution for this protozoa [7]. Specific tools for *E. histolytica* would better estimate of the occurrence of this parasite in the Manhiça district as the diagnostic tool used detects *E. histolytica/dispar*. The Manhiça district is a rural setting, where the children have more contact with soil, unimproved sanitary conditions including management of sewage, hygienic habits, and access to parasitic drugs [29].

*E. histolytica* was detected in an eleven months male child, underweighted and co-infected by *Cryptosporidium* spp. It is suggested that the male gender is more susceptible to infections due to androgens which reduce host immunity [30].

Less than 2% of the sample were co-infected. Previous studies conducted elsewhere in Africa also observed co-infections ranging from 0.25 to 43.4% [31–36]. It has been postulated that co-infections with *G. lamblia* can modulate the presence of symptoms in enteric infections. For example, co-infection between *G. lamblia* and rotavirus showed a fewer number of diarrhoea episodes compared to single infections by rotavirus [37]. On the other hand, previous studies indicated that *G. lamblia* was more common in non-diarrhoeal children [3, 7, 38], suggesting that symptoms in children infected by *G. lamblia* will be observed if there is another enteric pathogen that can contribute to a clinical profile. Further analysis needs to be done to evaluate the clinical characteristic of co-infections, including the presence of *G. lamblia*.

The caregiver’s education level was an independent risk factor for infection by *Cryptosporidium* spp. It was previously observed that a higher education level is directly associated with better hygiene habits and sanitary conditions. Therefore children with caregivers who are literate will be less susceptible to infection [10–12, 39, 40].

Undernutrition and infection by *Cryptosporidium* spp. share a similar geographic distribution [13]. The infection can cause inflammation and damage to the small intestine, leading to an undernourished profile and growth impairment [13]. An association between *G. lamblia* and nutritional status was not observed in this analysis. Although this relationship has been reported for chronic infection with *G. lamblia* [41]. However, we could not identify if children positive for *G. lamblia* had a chronic infection.

A high number of members in the children’s household was a risk factor for infection by *G. lamblia*. Little is known regarding this outcome, especially when protozoa infective-dose is considered; however, it is known that crowded environments contribute to parasites transmission [11, 12]. On the contrary, the relation between *Cryptosporidium* spp. and the number of household members was not observed. *G. lamblia* was more common in children without diarrhoea aged 12 to 59 months than in the ones with diarrhoea in the Global Enteric Multicenter Study (GEMS), which included Gambia, Mali, Mozambique, Kenya, India, Bangladesh, and Pakistan. Therefore, it seems that *G. lamblia* is more ubiquitous than *Cryptosporidium* spp., making the probability of a child to be infected by *G. lamblia* in a crowded environment much greater than by *Cryptosporidium* spp. [3].

A previous study conducted at HCN between 2012 and 2013, included children with undernourished, HIV, and diarrhoea, observed *G. lamblia* genotype B as the most common, which is related to anthroponomical transmission [18]. Previous results from HCN suggests that the absence of relation between animal contact and infection by *G. lamblia* in our sample may indicate the circulation of an anthroponomical genotype [18].

The drinking water source has been suggested as one of the routes for transmission of intestinal protozoa [14, 42, 43]. Transmission by water sources has been pointed mostly in developed countries outside Africa. Detection and notification are mostly reported by water suppliers in the outbreak context [9]. No outbreak was recorded during the survey period in the HCN, which can justify the absence of a relationship between drinking water source and infection by an intestinal protozoa.

The limitations in this analysis were: collection of a single stool sample from each participant, although it is recommended testing three samples for each individual, to avoid underestimation; and using a cross-sectional study design, which cannot measure causality between dependent and independent variables [44, 45]. However, we used a more sensitive diagnostic technique to minimize the underestimation of the true occurrence of intestinal protozoa in our samples [27, 28].

Our data indicate the presence of intestinal protozoa in 21.4% of the children with diarrhoea in HCN, however, 78.6% of the hospitalized children have unknown aetiology. Novel diagnostic tools such as multiplex techniques that identify a wider range of enteric pathogens should be considered to estimate the true burden of enteric pathogens in children with diarrhoea as well as co-infections and their clinical profiles especially in the presence of *G. lamblia* [4, 37].

## Conclusions

*Cryptosporidium* spp. and *Giardia lamblia* were the most common pathogenic intestinal protozoa detected in children with diarrhoea hospitalized in the *Hospital Central de Nampula*. The results obtained highlight the importance of further exploring the caregiver’s education level, children’s nutritional status for infections with *Cryptosporidium* spp., and living conditions, namely crowded households for infections with *G. lamblia* in children younger than five years.

## Data Availability

The dataset used and/or analysed during the current analysis are available from the corresponding author on reasonable request.

## Abbreviations

AOR: Adjusted odds ratio
CI: Confidence interval
DFG: Deutsche Forschungsgemeinschaft
EFINTD: European Foundation Initiate into African Research in Neglected Tropical Diseases
Gavi-HSS: The Vaccine Alliance Health System Strengthening
GEMS: Global Enteric Multicenter Study
ELISA: Enzyme-Linked Immunosorbent Assay
IBM SPSS: International Business Machines Corporation Statistical Package for the Social Science
INS: *Instituto Nacional de Saúde*
HCN: *Hospital Central de Nampula*
HIV: Human Immunodeficiency Virus
ViNaDia: *Vigilância Nacional de Diarreias*
WAZ: Weight-for-Age Z-score
WHO: World Health Organization
